# Fertility problems in males carrying an inversion of chromosome 10

**DOI:** 10.1515/med-2021-0240

**Published:** 2021-02-18

**Authors:** Xinyue Zhang, Qingyang Shi, Yanhong Liu, Yuting Jiang, Xiao Yang, Ruizhi Liu, Hongguo Zhang

**Affiliations:** Center for Reproductive Medicine and Center for Prenatal Diagnosis, First Hospital, Jilin University, 1 Xinmin Street, Chaoyang District, Changchun, Jilin Province, 130021, China

**Keywords:** chromosome 10, inversion, male infertility, genetic counselling

## Abstract

Chromosomal inversion is closely related to male infertility. Inversion carriers may produce abnormal gametes, which may lead to partial duplication/deletion of the embryonic chromosome and result in spontaneous abortion, a fetus with multiple anomalies, or birth of a malformed child. Genetic counselling remains challenging for these carriers in clinical practice. We report two male carriers with inversion of chromosome 10 and review 26 reported cases. In the first case, 46,XX,inv(10)(p13q22) of the fetal chromosome was found in prenatal diagnosis; this was inherited from the paternal side with 46XY,inv(10)(p13q22). Another case was a male carrier with inv(10)(q21.2q22.1). There have been 25 (89.3%) cases of pericentric inversion and three (10.7%) cases of paracentric inversion involving chromosome 10. Of 28 cases, nine were associated with pregestational infertility of the couples, while the other 19 cases were associated with gestational infertility of the couples or normozoospermia. The breakpoints at 10p15, 10p11, 10q11, and 10q21 were associated with pregestational infertility of the couples. The breakpoints at 10p15, 10p14, 10p13, 10p12, 10p11, 10q11, 10q21, 10q22, 10q23, 10q24, 10q25, and 10q26 were related to gestational infertility of the couples or normozoospermia. Although there is a high risk of infertility or recurrent miscarriages, carriers with inversion of chromosome 10 might produce healthy offspring. Natural pregnancy can be used as a choice for inversion carriers with recurrent spontaneous abortion.

## 1 Introduction

Male infertility is a complex multifactorial pathological condition with heterogeneity [1] and accounts for approximately 50% of infertile couples [2]. Genetic causes are responsible for approximately 15% of infertility in men [3]. Chromosomal disorders are considered to be an important genetic factor leading to defects of spermatogenesis. Chromosomal inversion and its breakpoint are closely related to male infertility [4,5,6].

Chromosomal inversion refers to the occurrence of a two-break event in a chromosome, and the segment rotates 180 degrees before reinserting [7]. However, inversion carriers may produce abnormal gametes through meiosis, which may lead to partial duplication/deletion of the embryonic chromosome. This then results in spontaneous abortion, a fetus with multiple anomalies, or birth of a malformed child [5]. With regard to chromosome 10 inversion, inv(10)(p15q24) has been reported in three generations of a family [8]. In paracentric inversion of chromosome 10 [inv(10)(q11.22q21.1)], the carriers have a normal phenotype, and no known gene is directly disrupted by the inversion [9]. Collinson et al. [10] reported that inv(10)(p11.2q21.2) was a benign variant. An increasing number of cases with inversion of chromosome 10 have been reported with development of clinical research. However, genetic counselling remains a challenge for chromosome 10 inversion carriers in clinical practice.

We report two male cases of chromosome 10 inversion. We also discuss the clinical fertility problems of men carrying chromosome 10 inversion.

## 2 Methods

This study was approved by the Ethics Committee of the First Hospital of Jilin University (No. 2019-300) and written informed consent was provided by each patient.

A 37-year-old man was phenotypically normal with average intelligence. His wife chose amniocentesis for prenatal diagnosis at 19 weeks of pregnancy because of advanced maternal age. Amniotic fluid cells were obtained through amniocentesis after written informed consent was obtained and collected by centrifugation. Amniocytes were inoculated in flasks using laboratory standards and cultured in carbon dioxide incubators for 12 days.

A 31-year-old man with a normal phenotype had a height of 169 cm and weight of 78.5 kg. Cytogenetic detection was performed for the man and his wife because his wife had two spontaneous abortions. After informed consent, peripheral blood was collected and chromosomal preparations were obtained from lymphocyte cultures. Cell harvesting was performed after the peripheral blood lymphocytes were cultured for 3 days. Giemsa staining of metaphase chromosomes was conducted according to the laboratory standard procedure. Twenty metaphases were counted and five karyotypes were analyzed. The couple was recalled to perform karyotype analysis because of abnormality of the fetal chromosome. Chromosomal analysis was performed as described in our previous study [11].

Papers on male chromosome 10 inversions were searched for using the PubMed database. The search keywords were “chromosome/inversion/male infertility” and “inversion/abortion.” We also analyzed a reference list that we created of papers that we had previously read. We included male adults with chromosome 10 inversion who were of a fertile age and excluded women and newborn carriers and those with bone marrow detection involving chromosome 10 inversion.

## 3 Results

### 3.1 Case description

#### 3.1.1 Case 1

**Figure 1:**
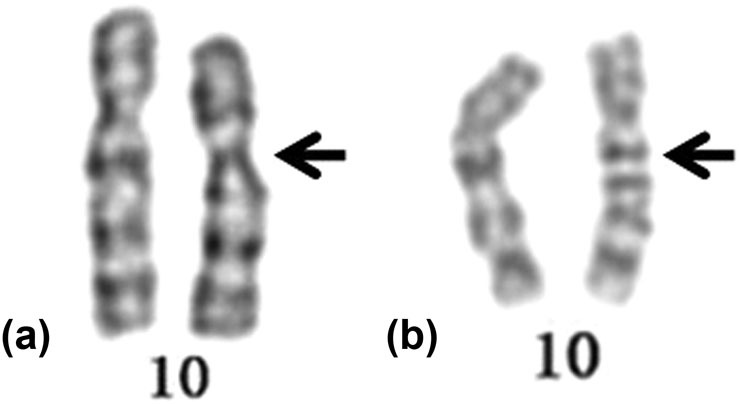
(a) Karyotype of the first case and (b) karyotype of the second case. The arrows indicate an abnormal chromosome 10.

The variant 46,XXinv(10)(p13q22) of the fetal chromosome was found in prenatal diagnosis. Further detection of the couple’s chromosomes showed that the fetal chromosomes were inherited from the father. The husband’s karyotype was 46,XY,inv(10)(p13q22) (Figure 1a) and his wife had a normal karyotype. They were a nonconsanguineous couple and the wife had no history of spontaneous abortion.

#### 3.1.2 Case 2

A karyotype result showed that the chromosome of the husband was 46,XY,inv(10)(q21.2q22.1) (Figure 1b) and his wife had a normal karyotype. His wife had a history of recurrent miscarriage. Unfortunately, no genetic testing was conducted for products of conception from the spontaneous abortions.

### 3.2 Review of the literature

After reviewing the literature, clinical findings and breakpoints in chromosome 10 inversion carriers were collected and summarized (Table 1). We found 26 carriers of chromosome 10 inversions. Including the current two cases, we found 25 (89.3%) cases of pericentric inversion and three (10.7%) cases of paracentric inversion associated with chromosome 10. Nine cases were associated with pregestational infertility of the couples, while the other 19 cases were associated with gestational infertility of the couples or normal fertility. The breakpoints at 10p15, 10p11, 10q11, and 10q21 were associated with pregestational infertility of the couples. The breakpoints at 10p15, 10p14, 10p13, 10p12, 10p11, 10q11, 10q21, 10q22, 10q23, 10q24, 10q25, and 10q26 were related to gestational infertility of the couples or normal fertility.

**Table 1:** Clinical findings in the couples with male partners carrying chromosome 10 inversions

Cases	Karyotype	Family history	Clinical findings	Reference
1	inv(10)(p15.2q11.22)	N/A	Hypogonadotropic hypogonadism	Helszer et al. [26]
2	inv(10)(p15.1q25.2)	Have inv(10) recombinant in two affected sibs	46,XY,inv(10)(p15.1q25.2) was found in the father and the healthy son	Roberts et al. [27]
3	inv(10)(p15q11)	N/A	Infertility	Teyssier et al. [28]
4	inv(10)(p15q24)	Observed in three generations of a family	One daughter with the inv(10) and trisomy 18. The other offspring with a recombinant (10) chromosome	Rodriguez et al. [8]
5	inv(10)(p14q21)	2 spontaneous abortions	N/A	Ghazaey et al. [29]
6	inv(10)(p13q22.3)	N/A	94.05% normal or balanced spermatozoa	Perrin et al. [30]
7	inv(10)(p13q23.1)	N/A	Non-iatrogenic azoospermic men; Sperm retrieval at surgery	Donker et al. [31]
8	inv(10)(p12q21)	N/A	Normozoospermia	Pylyp et al. [32]
9	inv(10)(p12q21)	3 spontaneous abortions	N/A	Husslein et al. [33]
10	inv(10)(p11.3;q21.2)	N/A	Azoospermia	Peschka et al. [34]
11	inv(10)(p11.22q21.1)	Recurrent abortions	Sterility	Groupe de Cytogénéticiens Français [35]
12	inv(10)(p11.21q21.2)	Recurrent fetal wastage	N/A	Fryns et al. [36]
13	inv(10)(p11.2q21.2)	Miscarriage	Severe oligozoospermia	Dul et al. [37]
14	inv(10)(p11.2q21.2)	Recurrent abortions	Sterility	Collinson et al. [10]
15	inv(10)(p11.2q21.2)	Recurrent pregnancy loss	N/A	Stephenson et al. [38]
16	inv(10)(p11.1q21.2)	Lack of conception	Normozoospermia	Olszewska et al. [39]
20	inv(10)(p11.2q21.2)	20 apparently unrelated families	No adverse clinical findings	Gilling et al. [21]
17	inv(10)(p11.2q21)	N/A	Severe oligozoospermia	Mierla et al. [40]
18	inv(10)(p11.2q21)	N/A	Infertility	Dana et al. [41]
19	inv(10)(p11.2q26.3)	Have a 2-year-old healthy daughter with 46,XX and a fetus with prominent facial dysmorphism	A paternal pericentric inversion	Chen et al. [42]
21	inv(10)(p11q21)	N/A	Oligospermia	Teyssier et al. [28]
22	inv(10)(p11q21)	Childless at age 34	Hypogonadism	de la Chapelle et al. [43]
23	inv(10)(p11q21.2)	Have normal infant born	Normozoospermia	Penso et al. [44]
24	inv(10)(p11q21)	N/A	Sterile male	Collinson et al. [10]
25	inv(10)(q11q26)	Familial inheritance	19 family members over three generations carry the same paracentric inversion	Venter et al. [45]
26	inv(10)(q11.22q21.1)	No family history	An inherited chromosome variant	Entesarian et al. [9]

N/A: not applicable.

## 4 Discussion

Inversion is one of the most common structural chromosomal balanced rearrangements. Although inversion carriers usually have a normal phenotype, the inverted chromosome region causes synaptic and recombinational problems during meiosis [12]. For men, inversion can disrupt spermatogenesis and lead to production of unbalanced spermatozoa through formation of an inversion loop [13]. Individuals who obtain these sperm will inevitably experience abortion, and the fetus can have delayed development, mental retrieval, or abnormal development of certain organ systems. Detection of spermatozoa from inversion carriers should be included in genetic counselling of infertile men to allow a personalized risk assessment [13]. However, there is a negligible risk of producing viable unbalanced offspring for paracentric inversion [14]. Therefore, appropriate genetic counselling for these carriers depends on the involved chromosomes and its breakpoints.

The current study identified two male inversion 10 carriers. In the first case, the husband’s wife had no history of spontaneous abortion and was pregnant with a fetus with chromosome 10 inversion. A newborn with a normal phenotype was delivered. The second case was a male carrier with paracentric inversion. His wife had two spontaneous abortions. We performed a literature search to review the clinical characteristics and provide appropriate genetic counselling for inversion 10 carriers. Twenty-six cases of chromosome 10 inversion are summarized in Table 1. According to Li et al. [15], male infertility can be divided into pregestational and gestational infertility of couples. Pregestational infertility is characterized by failure to produce a fertilized ovum. Gestational infertility is characterized by embryo loss after fertilization. Further analysis of the previous cases and our cases showed that the breakpoints at 10p15, 10p11, 10q11, and 10q21 were associated with pregestational infertility of the couples. All breakpoints on chromosome 10 were related to gestational infertility or normozoospermia.

With regard to pregestational infertility of the couples, the main clinical manifestations were severe oligozoospermia, azoospermia, infertility, and hypogonadism. To examine the role of breakpoints in chromosome 10 inversion in male infertility, we investigated whether certain genes on chromosome 10 are involved in spermatogenesis. The cAMP response element modulator gene (*CREM*), which is located on chromosome 10p11.21, may be responsible for activating several haploid germ cell-specific genes involved in the structure of the spermatozoon [16]. *CREM* is also thought to be important for mammalian spermatogenesis [17]. The TET oncogene family member 1 (*TET1*) gene has been mapped to chromosome 10q21.3. *TET1* has an important role in regulating related genes, which are involved in generation of gametes and meiosis [18]. Kim et al. [19] reported a breakpoint at 10q24 in cases of impaired spermatogenesis and recurrent abortion. In the case of gestational infertility in couples, the main clinical aspects are normozoospermia, spontaneous abortions, and familial inheritance. Although some cases with inversion of chromosome 10 show normal fertility, these carriers have a higher reproductive risk. For structural reorganization carriers, the mechanism of chromosomal abnormality affecting spermatogenesis includes the following: (1) an interchromosomal effect increases the risk of numerical chromosomal abnormalities in the gametes, (2) disturbance of chromosomal pairing, synapsis, and recombination during meiosis, (3) DNA fragmentation in spermatozoa and activation of apoptosis, and (4) interference of specific gene function at the breakpoint [4,20]. However, Young et al. [7] reported that infertile carriers with chromosomal inversions are not susceptible to an interchromosomal effect. Therefore, the exact mechanism of chromosomal abnormality affecting spermatogenesis requires further study.

Notably, inv(10)(p11.2q21.2) was once considered as a benign variant [10,21]. Table 1 shows that some cases of inv(10)(p11.2q21.2) showed recurrent spontaneous abortion. Moreover, polymorphic variants in chromosomes probably play a significant role in infertility [22]. Therefore, more attention should be paid to this inverted chromosome in genetic counselling.

For inversion carriers experiencing recurrent pregnancy loss, preimplantation genetic diagnosis is considered as part of clinical management, which can improve the pregnancy rate and reduce the abortion rate [23,24] However, preimplantation genetic diagnosis involves high additional costs and has potential complications for patients. Furthermore, the benefits of preimplantation genetic diagnosis to patients have not been confirmed [25]. Clinical physicians should pay attention to obtaining good reproductive results through natural pregnancy in genetic counselling.

In this study, we report two male carriers with inversion of chromosome 10 and review 26 reported cases. Despite the high risk of infertility and recurrent miscarriages, carriers of chromosome 10 inversion might be able to produce healthy offspring. Natural pregnancy can be used as a choice for carriers of chromosome 10 inversion with recurrent spontaneous abortion.
